# Fear of COVID‐19 Scale (FCV‐19S) across countries: Measurement invariance issues

**DOI:** 10.1002/nop2.855

**Published:** 2021-03-21

**Authors:** Chung‐Ying Lin, Wen‐Li Hou, Mohammed A. Mamun, José Aparecido da Silva, Yunier Broche‐Pérez, Irfan Ullah, Akihiro Masuyama, Koubun Wakashima, Mélody Mailliez, Arnaud Carre, Yu‐Pin Chen, Kun‐Chia Chang, Yi‐Jie Kuo, Paolo Soraci, Damian Scarf, Anders Broström, Mark D. Griffiths, Amir H. Pakpour

**Affiliations:** ^1^ Institute of Allied Health Sciences College of Medicine National Cheng Kung University Hospital National Cheng Kung University Tainan Taiwan; ^2^ College of Nursing Kaohsiung Medical University Kaohsiung Taiwan; ^3^ Department of Medical Research Kaohsiung Medical University Hospital Kaohsiung Taiwan; ^4^ CHINTA Research Bangladesh (Centre for Health Innovation, Networking, Training, Action and Research ‐ Bangladesh) Savar, Dhaka Bangladesh; ^5^ Unit of Psychobiology University of São Paulo Sao Paulo Brazil; ^6^ Psychology Department Universidad Central “Marta Abreu” de Las Villas Km 5 ½ Santa Clara Cuba; ^7^ Kabir Medical College Gandhara University Peshawar Pakistan; ^8^ Faculty of Psychology Iryo Sosei University Iwaki City Japan; ^9^ Graduate School of Education Tohoku University Sendai‐city Japan; ^10^ ISAE‐SUPAERO (Institut Supérieur de l'aéronautique et de l'espace) University of Toulouse Université Fédérale de Toulouse Midi‐Pyrénées) Toulouse France; ^11^ LIP/PC2S University of Savoie Mont Blanc University of Grenoble Alpes Chambéry France; ^12^ Department of Orthopedic Surgery Wan Fang Hospital Taipei Medical University Taipei Taiwan; ^13^ Department of Orthopedic Surgery School of Medicine College of Medicine Taipei Medical University Taipei Taiwan; ^14^ Jianan Psychiatric Center Ministry of Health and Welfare Tainan Taiwan; ^15^ Department of Natural Biotechnology NanHua University Chiayi Taiwan; ^16^ Group Cognitive Behavioral Psychology Association Rome Italy; ^17^ Department of Psychology University of Otago Dunedin New Zealand; ^18^ Department of Nursing School of Health and Welfare Jönköping University Jönköping Sweden; ^19^ Department of Clinical Neurophysiology University Hospital Linköping Linköping Sweden; ^20^ International Gaming Research Unit Psychology Department Nottingham Trent University Nottingham UK; ^21^ Social Determinants of Health Research Center Research Institute for Prevention of Non‐Communicable Diseases Qazvin University of Medical Sciences Qazvin Iran

**Keywords:** assessment, COVID‐19, cross‐cultural, differential item functioning, FCV‐19S, Fear of COVID‐19, Rasch analysis

## Abstract

**Aim:**

The threats of novel coronavirus disease 2019 (COVID‐19) have caused fears worldwide. The Fear of COVID‐19 Scale (FCV‐19S) was recently developed to assess the fear of COVID‐19. Although many studies found that the FCV‐19S is psychometrically sound, it is unclear whether the FCV‐19S is invariant across countries. The present study aimed to examine the measurement invariance of the FCV‐19S across eleven countries.

**Design:**

Cross‐sectional study.

**Methods:**

Using data collected from prior research on Bangladesh (*N* = 8,550), United Kingdom (*N* = 344), Brazil (*N* = 1,843), Taiwan (*N* = 539), Italy (*N* = 249), New Zealand (*N* = 317), Iran (*N* = 717), Cuba (*N* = 772), Pakistan (*N* = 937), Japan (*N* = 1,079) and France (*N* = 316), comprising a total 15,663 participants, the present study used the multigroup confirmatory factor analysis (CFA) and Rasch differential item functioning (DIF) to examine the measurement invariance of the FCV‐19S across country, gender and age (children aged below 18 years, young to middle‐aged adults aged between 18 and 60 years, and older people aged above 60 years).

**Results:**

The unidimensional structure of the FCV‐19S was confirmed. Multigroup CFA showed that FCV‐19S was partially invariant across country and fully invariant across gender and age. DIF findings were consistent with the findings from multigroup CFA. Many DIF items were displayed for country, few DIF items were displayed for age, and no DIF items were displayed for gender.

**Conclusion:**

Based on the results of the present study, the FCV‐19S is a good psychometric instrument to assess fear of COVID‐19 during the pandemic period. Moreover, the use of FCV‐19S is supported in at least ten countries with satisfactory psychometric properties.

## 1 INTRODUCTION

The threats and consequences of the novel coronavirus disease 2019 (COVID‐19) to individual's health and related aspects have been investigated in many different ways, including their psychological health and behaviours from individual and government perspectives (Lin & Cheng, 2020; Rieger, 2020; Shrivastava & Shrivastava, 2020). In addition to the risks of death and serious consequences due to COVID‐19 infection, scholars and healthcare professionals have identified the need to assist different populations in tackling mental health difficulties (Holmes et al., 2020; Islam et al., 2020; Usman et al., 2020). More specifically, individuals may have elevated psychological distress and perform inappropriate life‐threatening behaviours induced by the elevated distress due to the COVID‐19 pandemic (Dsouza et al., 2020; Griffiths & Mamun, 2020; Lin, 2020; Mamun & Ullah, 2020). In order to respond to the need of assessing mental health issues, several research teams have developed different instruments to understand the psychological response to COVID‐19 (Ahorsu, Lin, Imani, et al., 2020; Ahorsu, Lin, & Pakpour, 2020; Lee, 2020a, 2020b; Taylor et al., 2020). These instruments include the: (a) five‐item Coronavirus Anxiety Scale (CAS) (Lee, 2020a), (b) four‐item Obsession with COVID‐19 Scale (OCS) (Lee, 2020a), (c) 36‐item COVID Stress Scale (CSS) (Taylor et al., 2020) and (d) seven‐item Fear of COVID‐19 Scale (FCV‐19S) (Ahorsu, Lin, Imani, et al., 2020; Ahorsu, Lin, & Pakpour, 2020). Moreover, Ransing et al.   (2020) conducted a rapid review to summarize the features of these four instruments. Ransing et al. (2020) indicated that one of the most important issues for these instruments was the need to translate, culturally adapt, assess and validate the existing instruments to achieve the maximum utility.

Pakpour, Griffiths, Chang, et al. (2020) responded to Ransing et al. (2020) and demonstrated that the FCV‐19S had strong features in its brevity with satisfactory psychometric properties shown in different language versions (Pakpour, Griffiths, & Lin, et al., 2020). Indeed, in 3 months of the original Persian FCV‐19S being published (Ahorsu, Lin, Imani, et al., 2020; Ahorsu, Lin, & Pakpour, 2020), the scale had been validated in English [in the UK (Harper et al., 2020), in New Zealand (Winter et al., 2020)], Arabic (Alyami et al., 2020), Bangla (Sakib et al., 2020) Italian (Soraci et al., 2020), Hebrew (Bitan et al., 2020), Russian (Reznik et al., 2020), Turkish (Satici et al., 2020), Chinese (Pakpour, Griffiths, Chang, et al., 2020), Urdu, Malay (Pang et al., 2020), Brazilian Portuguese (Abad et al., 2020), Cuban Spanish (Broche‐Pérez et al., 2020) and Greek (Tsipropoulou et al., 2020).

All the different language versions of FCV‐19S were found to have satisfactory psychometric properties, including internal consistency (*α* or *ω* = 0.82 for Persian; 0.88 for British English; 0.88–0.89 for New Zealand English; 0.88 for Arabic; 0.87 for Bangla; 0.87 for Italian; 0.77–0.86 for Hebrew; 0.81 for Russian; 0.85 for Turkish; 0.93 for Chinese; 0.89 for Malay; 0.87 for Cuban Spanish; and 0.87 for Greek); test‐retest reliability (*r* or ICC = 0.72 for Persian; 0.87 for Bangla; and 0.97 for Malay); concurrent validity (absolute *r* = .42–.51 for Persian; 0.31 for British English; 0.31–0.40 for New Zealand English; 0.66 for Arabic; 0.41 for Bangla; 0.65–0.70 for Italian; 0.21–0.46 for Hebrew; 0.73 for Brazilian Portuguese; and 0.47–0.71 for Greek); and construct validity (supported unidimensional or two‐factor structure in either confirmatory factor analysis or exploratory factor analysis across all language versions). Although most of the studies conducted to date have reported a unidimensional structure, a couple of studies have reported a two‐factor structure (Pakpour, Griffiths, & Lin, et al., 2020; Ransing et al., 2020).

Although a two‐factor structure has been proposed and tested, the two‐factor structure does not have the theoretical background to support it (Pakpour, Griffiths, Chang, et al., 2020; Pakpour, Griffiths, & Lin, et al., 2020). More specifically, the original FCV‐19S (Ahorsu, Lin, Imani, et al., 2020; Ahorsu, Lin, & Pakpour, 2020) was developed using Protection Motivation Theory (Rogers, 1975), and was identified as having a single‐factor structure using exploratory factor analysis (Ahorsu, Lin, Imani, et al., 2020; Ahorsu, Lin, & Pakpour, 2020) with the single‐factor structure verified in confirmatory factor analysis (Alyami et al., 2020; Pakpour, Griffiths, Chang, et al., 2020; Sakib et al., 2020; Satici et al., 2020; Soraci et al., 2020; Tsipropoulou et al., 2020). Therefore, the present authors believe that the FCV‐19S should have a single‐factor structure across different language versions. However, at the time of writing, no studies have examined the measurement invariance of the FCV‐19S to verify whether its factor structure is equivalent across different subgroups, including different language versions. Additionally, it is still unclear whether different subgroups (e.g. different ethnic populations, different genders and different age groups) interpret the FCV‐19S with similar considerations. Therefore, this is an important missing aspect in the extant literature and the present authors attempted to answer the research question of whether individuals from different countries interpret FCV‐19S items similarly.

Consequently, measurement invariance is an important issue for an instrument to satisfy the aforementioned question (i.e. whether different subgroups interpret FCV‐19S items similarly). If the psychometric testing on measurement invariance supports the invariance across subgroups, this indicates that individuals in the subgroups interpret the instrument concept and content (e.g. FCV‐19S in the present study) in the same way (Limbers et al., 2008; Lin et al., 2013). Moreover, with the use of measurement invariance, the underlying concept can be compared in a more accurate way than using the comparison with observed scores (Vandenberg & Lance, 2000). For example, some studies have used observed quality of life (QoL) scores (i.e. summing up all the item scores) to compare the quality of life between groups (Bodur & Cingil, 2009; Su et al., 2013, 2014). However, this practice of comparisons does not consider any measurement errors or measurement weights. In other words, such comparisons using observed QoL scores are not accurate. In contrast, comparisons using the latent scores with the consideration of measurement invariance, instead of the observed scores, tackle the aforementioned measurement issues (Lin et al., 2016). Therefore, testing measurement invariance is important for an instrument to help healthcare providers and researchers meaningfully compare an underlying concept (e.g. fear of COVID‐19 in the FCV‐19S) between subgroups.

The present study asserts that the FCV‐19S should be evaluated for its measurement invariance in three aspects: different ethnicity, gender and age (i.e. children aged below 18 years, young to middle‐aged adults aged between 18 and 60 years, and older people aged over 60 years). Cultural differences and the different actions and policies made by different governments internationally may make different ethnic populations respond differently to FCV‐19S items. For example, Western people as compared with Eastern people are prone to individualism (Dubois & Beauvois, 2005; Harkness et al., 2000). Therefore, Western people intend to respect personal freedom whenever such freedom does not break the laws. In contrast, Eastern people are more collectivist (King & Bond, 1985; Lin & Tsai, 2016; Tsai et al., 2015) and care more about the harmony in the community. Therefore, Eastern people may be more likely to perform behaviours that the society wishes even though such behaviours are not required by the legislation. As a result, Rieger (2020) found that a sample of European students intended not to wear mask if there was no legislation.

The policies implemented by different governments internationally reflect the different levels of awareness or different ways countries assess the risk of COVID‐19 and how they interpret the possible consequences. Although the different policies across countries are not necessarily culturally different or ethnically different per se, the policies may influence residents' psychological state. Subsequently, the residents in these different countries may have different interpretations of items in the FCV‐19S. Taking Iranian and Taiwanese governments as examples, both governments adopted universal policies (e.g. border control, disseminating useful COVID‐19 information such as preventive COVID‐19 infection behaviours through different social media platforms) during the COVID‐19 outbreak. However, the Taiwanese government as compared with the Iranian government had a much quicker response to the COVID‐19 pandemic and the figures of COVID‐19 infected cases and deaths are very different between Iran and Taiwan (Chen, Jyan, et al., 2020; Lin & Cheng, 2020).

With respect to gender, prior evidence has shown that females (as compared with males) tend to be more sensitive to stress and, therefore, usually have higher levels of fear when encountering various life events (Tolin & Foa, 2006; Vlassoff, 2007). Therefore, it is not known whether males and females interpret items in the FCV‐19S in the same way. Similarly, recent evidence has shown that older people are more vulnerable to COVID‐19 infection and usually have a more serious prognosis than younger cohorts (Dariya & Nagaraju, 2020; Moccia et al., 2020). Therefore, relative to older people, younger people may not be as aware of the seriousness of COVID‐19 and have little in the way of a psychological response to COVID‐19.

In order to fully understand the interpretation of FCV‐19S items among different ethnic populations, different genders and different age groups (children, young to middle‐aged adults and older people), the present study used data derived from ten countries to examine the measurement invariance of the FCV‐19S. The single‐factor structure of the FCV‐19S was re‐examined in the large sample from a diverse cultural background. More specifically, the present study compared the FCV‐19S scores between ten countries comprising Bangladesh, United Kingdom, Brazil, Taiwan, Italy, New Zealand, Iran, Cuba, Pakistan and Japan. Additionally, the FCV‐19S scores between genders and age groups were assessed.

## 2 METHOD

### 2.1 Participants and recruitment procedure

The present study included the datasets from ten countries that have validated the FCV‐19S in their respective countries. A short sampling description is given herewith, details can be found in the original papers (Abad et al., 2020; Broche‐Pérez et al., 2020; Chang, Hou, et al., 2020; Harper et al., 2020; Mailliez et al., 2021; Masuyama et al., 2020; Pakpour, Griffiths, Chang, et al., 2020; Sakib et al., 2020; Soraci et al., 2020; Winter et al., 2020). More specifically, all the participants used in the present study were recruited through convenience sampling. Some were recruited using online surveys and some were recruited using paper‐based (offline) surveys because most of the validations were carried out independently by different research teams and the respective teams had different resources in the different countries. However, there was no serious bias in using the two types of survey data collection and there is prior evidence showing that online and offline surveys are measurement invariant (Martins, 2010). All the study designs were cross‐sectional. Moreover, general populations were the target sample in most of the countries (Table 1). Table 1 also reports the data collection period for each country and a related figure concerning COVID‐19 infection at the time of the study.

**TABLE 1:** Study characteristics sample

Country	Reference	Target sample	Sampling method	Data collection period	Government reaction toward COVID‐19	COVID‐19 condition during data collection period
Bangladesh	Sakib et al., 2020	General population (*N* = 8,550)	Online convenience sampling; Cross‐sectional design	April 2020	In March—education facilities closed, border screening, social distancing In April—Office holiday, suspicion of public transport, public gathering restrictions	330 total confirmed cases and 112 deaths with clusters of cases transmission as reported on 10 April
United Kingdom	Harper et al., 2020	General population (*N* = 344)	Online convenience sampling; Cross‐sectional design	March 2020	National lockdown was implemented on March 23 (all but essential services closed including shops, businesses and educational establishments)	By April 1st, 29,474 cases and 2,352 deaths.
Brazil	Abad et al. 2020	General Population (*N* = 1,843)	Online convenience sampling; Cross‐sectional design	April 2020	Social isolation and distancing; hygiene practices; school closures, strict regulations for events and public places; quarantine of infected peoples; closing non‐essential businesses	April 30th 6,006 deaths due to COVID‐19 87,187 people diagnosed with the disease. August 18th 1,352 deaths in the last 24 hr; 47,784 confirmed cases in the last 24 hr; 109,888 deaths; 3,407,354 confirmed cases.
Taiwan	Chang, Hou, et al., (2020); Pakpour, Griffiths, Chang, et al. (2020)	People with mental illness and older people (*N* = 539)	Convenience sampling; Cross‐sectional design	Between March and May 2020	Infection control policies implemented in late January, 2020 Strict regulations for events and public places	Confirmed cases: 442; Deaths: 7
Italy	Soraci et al., 2020	General population (*N* = 249)	Convenience sampling; Cross‐sectional design	March 2020	Blocked Chinese passengers on 30 January 2020 State of Emergency declared on 31 January 2020 Prohibition of access and removal in the municipalities with COVID‐19 outbreak implemented on 23 February 2020	Confirmed cases: 241,819 Deaths: 34,869
New Zealand	Winter et al., 2020 (data not published)	General population (*N* = 317)	Convenience sampling; Cross‐sectional design	Between March and May 2020	State of National Emergency declared on 25 March 2020	Confirmed cases: 2060 Deaths: 26
Iran	Ahorsu, Lin, Imani, et al., 2020	General population (*N* = 717)	Convenience sampling; Cross‐sectional design	March 2020	Infection control policies implemented in late February 2020	Confirmed cases: 7,161; Deaths: 237
Cuba	Broche‐Pérez et al., 2020	General population (*N* = 772)	Convenience sampling; Cross‐sectional design	Between April and May 2020	The Cuban government presents the action plan against COVID‐19 (January 2020) Mandatory use of facial mask (March 2020) Strict lockdown in areas with more than 10 confirmed cases (March 2020) Isolation of all suspected cases in specialized centres (March 2020) Closure of international borders (March 2020) Strict regulations for events and public places (March 2020)	Confirmed cases: 2,588 Deaths: 84
Pakistan	Ullaha et al., (n.d.).	General population (*N* = 937)	Convenience sampling; Cross‐sectional design	May 2020	Strict lockdown implemented on 22 March 2020 Smart lockdown implemented on 13 June 2020	Confirmed cases: 277,402 Deaths: 5,924
Japan	Masuyama et al., 2020 Wakashima et al., 2020	Adolescents and general population (*N* = 1,079)	Online convenience sampling; Cross‐sectional design	April 2020	National State of Emergency declared on 16 April 2020	Confirmed cases: 14,088 Deaths: 415
French	Mailliez et al. (under review)	General population (*N* = 316)	Convenience sampling, Cross‐sectional design	Between April and May 2020	Partial lockdown for sick people on 23 February 2020 Demonstration over 5,000 people is banned on 29 February 2020 Lockdown for general population between March 17 and May 11	Confirmed cases: 139,519 Deaths: 26,643

### 2.2 Measures

#### 2.2.1 Fear of COVID‐19 Scale (FCV‐19S)

The seven‐item FCV‐19S was developed to quickly assess individuals' fear towards COVID‐19 (Ahorsu, Lin, Imani, et al., 2020; Ahorsu, Lin, & Pakpour, 2020). Responding to items on a five‐point Likert scale (1 = strongly disagree; 5 = strongly agree), the FCV‐19S has been found to be psychometrically sound in assessing fear of COVID‐19 in different populations, including different ethnic groups (Alyami et al., 2020; Pakpour, Griffiths, Chang, et al., 2020; Pang et al., 2020; Sakib et al., 2020; Satici et al., 2020; Soraci et al., 2020; Tsipropoulou et al., 2020) and various vulnerable groups (Pakpour, Griffiths, Chang, et al., 2020). An example item in the FCV‐19S is “*I cannot sleep because I'm worrying about getting coronavirus‐19*”. A higher level of fear toward COVID‐19 is indicated by the higher FCV‐19S score. Moreover, different language versions of the FCV‐19S used in the present study have been validated (Alyami et al., 2020; Chang, Hou, et al., 2020; Pakpour, Griffiths, Chang, et al., 2020; Sakib et al., 2020; Satici et al., 2020; Soraci et al., 2020; Tsipropoulou et al., 2020).

### 2.3 Data analysis

The participants' age, gender distribution (male, female, and other), and FCV‐19S scores were first analysed using descriptive statistics for each country. Item properties of the seven FCV‐19S items were then examined using skewness, kurtosis (to check normal distribution of responses for each item), item difficulty (with the use of Rasch analysis), item fit (including information‐weighted fit mean square [MnSq] and outlier‐sensitive fit MnSq; where value between 0.5 and 1.5 indicates good fit) (Lin et al., 2019) factor loadings (derived from confirmatory factor analysis [CFA]) and item‐total correlations. The entire FCV‐19S scale properties were assessed using internal consistency, CFA and Rasch analysis. For internal consistency, Cronbach's *α* with a value >0.7 indicates satisfactory (Lee et al., 2016); for CFA, fit indices of comparative fit index (CFI) and Tucker‐Lewis index (TLI) > 0.9 with root mean square error of approximation (RMSEA) and standardized root mean square residual (SRMR) <0.08 indicate satisfactory (Lin et al., 2017); for Rasch analysis, item and person separation reliability >0.7 with item and person separation index >2 indicate satisfactory (Lin et al., 2019).

Differential item functioning (DIF) based on Rasch analysis was conducted to examine whether different interpretations of the FCV‐19S item content occurred across countries, gender (male and female) or age groups (children aged below 18 years, young to middle‐aged adults aged between 18 and 60 years and older people aged above 60 years). A substantial DIF is defined as a DIF contrast >0.5 (Lin et al., 2019). Measurement invariance was further tested using multigroup CFA to examine whether participants from different countries, different gender participants (male and female), and participants with different ages (children aged below 18 years, young to middle‐aged adults aged between 18 and 60 years, and older people aged above 60 years) interpret the entire FCV‐19S similarly. In the multigroup CFA, several nested models were compared. More specifically, configural models across countries, gender and age groups were first carried out to examine whether different aggregated subgroups of participants confirm the single‐factor structure of the FCV‐19S. Then, CFA models with factor loadings constrained equally across subgroups were constructed and compared with the configural models to examine whether different subgroups shared the same factor loadings. Finally, CFA models with factor loadings and item intercepts constrained equally across subgroups were constructed and compared with the models with factor loadings constrained equally to examine whether different subgroups shared the same item intercepts. ΔCFI > −0.01, ΔRMSEA < 0.01 and ΔSRMR < 0.01 support the full measurement invariance in every two nested models' comparisons (Lin et al., 2019). However, if the full measurement invariance was not achieved, partial invariance was tested using the process of relaxing factor loadings or item intercepts in the constrained models. Moreover, the data relating to “other” gender was not used for DIF or multigroup CFA because there were only 27 participants reporting their gender as other. Given the huge difference in sample sizes (27 “other” gender, 7,723 male gender, and 8,363 female gender), carrying out invariance testing on such a small sample size would be problematic.

A model with structural equation modelling (SEM) was then constructed to examine the associations between age, gender, and fear of COVID‐19. In the SEM model, young to middle‐aged adults aged between 18 and 60 years and being male were reference groups. All the statistical analyses were performed using SPSS 24.0 (IBM corp.), WINSTEPS 4.1.0 (winsteps.com), and lavaan package (https://lavaan.ugent.be/tutorial/index.html) in the R software.

## 3 RESULTS

### 3.1 Demographics and the FCV‐19S score across countries

Table 2 presents the age, gender and FCV‐19S scores across the ten countries. The Bangladesh cohort had the most participants (*N* = 8,550) and the Italian cohort had the fewest participants (*N* = 249). Moreover, the participants from New Zealand were the youngest (mean age = 19.7 years; *SD* = 3.1) and those from Taiwan were the oldest (mean age = 53.3 years; *SD* = 14.9). In regard to the gender distribution, only the Japan and Bangladesh cohorts had more males (Japan: 56.6%; Bangladesh: 56.0%) than females. All the other countries had fewer male participants (8.0% to 49.0%). Furthermore, participants from Iran had the highest levels of fear of COVID‐19 (mean score of FCV‐19S = 3.92; *SD* = 0.64) and those from New Zealand had the lowest levels of fear (mean score of FCV‐19S = 2.02; *SD* = 0.80).

**TABLE 2:** Participant characteristics and Fear of COVID‐19 Scale (FCV‐19S) scores

	Country										
	Bangladesh (*N* = 8,550)	UK (*N* = 344)	Brazil (*N* = 1,843)	Taiwan (*N* = 539)	Italy (*N* = 249)	NZ (*N* = 317)	Iran (*N* = 717)	Cuba (*N* = 772)	Pakistan (*N* = 937)	Japan (*N* = 1,079)	French (*N* = 316)
Age (years); *M* (*SD*)	26.5 (9.1)	34.5 (12.0)	36.2 (12.8)	53.3 (14.9)	34.5 (12.2)	19.7 (3.1)	31.1 (12.6)	35.9 (14.6)	25.8 (11.8)	27.6 (19.6)	35.57 (14.3)
Gender (male); *N* (%)	4,790 (56.0)	165 (48.0)	367 (19.9)	264 (49.0)	20 (8.0)	110 (34.6)	301 (42.0)	203 (26.3)	400 (42.7)	611 (56.6)	42 (13.3)
F1; *M* (*SD*)	3.62 (1.04)	3.25 (1.13)	3.49 (1.22)	2.99 (1.42)	3.44 (1.08)	2.50 (1.15)	3.48 (1.14)	3.47 (1.68)	2.97 (1.06)	3.99 (1.13)	2.99 (1.16)
F2; *M* (*SD*)	3.52 (1.06)	3.23 (1.20)	3.17 (1.29)	2.49 (1.37)	2.94 (1.27)	2.43 (1.18)	4.01 (0.84)	4.10 (1.39)	2.99 (1.16)	3.38 (1.23)	2.83 (1.28)
F3; *M* (*SD*)	2.50 (1.13)	1.79 (0.94)	1.56 (0.94)	2.05 (1.15)	1.50 (0.94)	1.59 (0.83)	3.76 (0.88)	1.61 (1.25)	2.07 (1.10)	1.85 (1.01)	1.54 (0.88)
F4; *M* (*SD*)	2.93 (1.23)	2.64 (1.30)	2.95 (1.47)	2.60 (1.40)	2.42 (1.27)	1.87 (1.03)	4.24 (0.90)	3.04 (1.77)	2.67 (1.24)	3.88 (1.21)	2.17 (1.31)
F5; *M* (*SD*)	3.53 (1.07)	3.25(1.25)	3.01 (1.34)	2.61 (1.30)	2.94 (1.27)	2.57 (1.28)	3.53 (1.07)	3.65 (1.56)	3.11 (1.18)	3.03 (1.26)	3.00 (1.40)
F6; *M* (*SD*)	2.41 (1.11)	1.94(1.09)	1.79 (1.08)	2.08 (1.16)	1.56 (0.88)	1.56 (0.85)	4.11 (0.81)	2.15 (1.57)	2.01 (1.14)	1.77 (0.97)	1.58 (0.97)
F7; *M* (*SD*)	2.88 (1.24)	1.94(1.12)	2.12 (1.33)	2.11 (1.19)	2.10 (1.28)	1.64 (0.91)	4.27 (0.75)	2.89 (1.76)	2.35 (1.22)	1.89 (1.10)	1.72 (1.08)
FCV‐19S; *M* (*SD*)	3.05 (0.85)	2.58 (0.89)	2.58 (0.95)	2.42 (1.04)	2.41 (0.86)	2.02 (0.80)	3.92 (0.64)	2.99 (1.06)	2.59 (0.89)	2.83 (0.81)	2.26 (0.89)

Note

F1 = I am most afraid of coronavirus‐19.

F2 = It makes me uncomfortable to think about coronavirus‐19.

F3 = My hands become clammy when I think about coronavirus‐19.

F4 = I am afraid of losing my life because of coronavirus‐19.

F5 = When watching news and stories about coronavirus‐19 on social media, I become nervous or anxious.

F6 = I cannot sleep because I'm worrying about getting coronavirus‐19.

F7 = My heart races or palpitates when I think about getting coronavirus‐19.

Abbreviations: FCV‐19S, Fear of COVID‐19 Scale; NZ, New Zealand.

### 3.2 Item properties of the FCV‐19S across countries

Table 3 further demonstrates the item properties of the FCV‐19S. All the seven items were nearly normally distributed (skewness = −0.61 to 0.73; kurtosis = −1.27 to −0.46) with the difficulty ranged between −0.88 and 1.01. All the items had satisfactory fit statistics (infit MnSq = 0.88–1.13; outfit MnSq = 0.86–1.13), strong factor loadings (0.636–0.747) and high item‐total correlations (0.61–0.68). The entire FCV‐19S scale properties were also satisfactory as demonstrated by the very good internal consistency (*α* = 0.87), excellent fit statistics in the CFA (CFI = 0.983, TLI = 0.974, RMSEA = 0.076 and SRMR = 0.059), and promising separation reliability and index in the Rasch analysis (item separation reliability = 1.00, item separation index = 73.32, person separation reliability = 0.84, and person separation index = 2.27) (Table 4).

**TABLE 3:** Item properties of the Fear of COVID‐19 Scale (FCV‐19S)

	Skewness	Kurtosis	Difficulty	Infit MnSq	Outfit MnSq	Factor loadings	Item‐total correlation
F1	−0.61	−0.46	−0.91	1.07	1.13	0.636	.61
F2	−0.57	−0.65	−0.74	1.01	1.03	0.666	.63
F3	0.68	−0.56	1.01	0.91	0.90	0.712	.66
F4	−0.06	−1.27	−0.10	1.13	1.10	0.705	.65
F5	−0.58	−0.73	−0.63	0.94	0.94	0.701	.66
F6	0.73	−0.52	0.98	0.88	0.86	0.727	.67
F7	0.27	−1.25	0.39	1.02	1.01	0.747	.68

Abbreviations: Infit MnSq, information‐weighted fit mean square; Outfit MnSq, outlier‐sensitive fit mean square.

**TABLE 4:** Structure fit of the Fear of COVID‐19 Scale (FCV‐19S)

Fit testing	Value
Internal consistency (Cronbach's *α*)	0.87
Confirmatory factor analysis	
χ^2^ (*df*)/*p*‐value	1,281.676 (14)/<.001
Comparative fit index	0.983
Tucker‐Lewis index	0.974
Root mean square error of approximation	0.076
Standardized root mean square residual	0.059
Rasch analysis	
Item separation reliability	1.00
Item separation index	73.32
Person separation reliability	0.84
Person separation index	2.27

### 3.3 Measurement invariance and factor loading findings for the FCV‐19S

Differential item functioning contrasts across different countries, gender and age groups are presented in Table 5. Apparently, most of the FCV‐19S items displayed substantial DIF across the ten countries of Bangladesh, United Kingdom, Brazil, Taiwan, Italy, New Zealand, Iran, Cuba, Pakistan and Japan. However, no substantial DIF items were observed across gender (DIF contrasts = −0.24 and 0.16). About the age groups, four items (F1 and F7 between children and young to middle‐aged adults; F4 between children and young to middle‐aged adults and between young to middle‐aged adults and older people; F6 between children and young to middle‐aged adults and between children and older people) displayed DIF. Similar conditions were shown in the measurement invariance testing using the multigroup CFA. Only partial invariance was supported for the FCV‐19S across countries (with the relaxed factor loadings of items F2 and F3; relaxed item intercepts of items F1 and F3 to F6). About gender and age groups, full invariance was supported for the FCV‐19S. However, the ∆CFI, ∆RMSEA and ∆SRMR were larger in the multigroup CFA across age groups than in the multigroup CFA across gender (Table 6).

**TABLE 5:** Differential item functioning (DIF) contrast across gender and country

	DIF Contrast						
	F1	F2	F3	F4	F5	F6	F7
Country							
1 versus 2	**−1.02**	−0.11	**0.65**	**−1.78**	0.40	**0.69**	**1.11**
1 versus 3	**−0.83**	0.21	0.17	**−1.46**	**0.74**	**0.63**	0.38
1 versus 4	−0.41	0.15	**−0.52**	**−1.00**	0.40	0.23	**0.64**
1 versus 5	**−0.82**	**−0.63**	**0.81**	**−1.24**	0.07	**1.03**	**0.85**
1 versus 6	−0.30	0.04	−0.32	**−1.52**	**0.54**	0.01	**0.93**
1 versus 7	**−0.97**	−0.01	**0.62**	**−1.80**	**0.71**	**0.71**	**0.67**
1 versus 8	**−2.74**	**−0.69**	**1.08**	**−1.00**	**−1.02**	**1.89**	**2.00**
1 versus 9	**−1.03**	**1.35**	**−1.17**	**−1.50**	**0.87**	0.18	**1.20**
1 versus 10	**−1.28**	−0.18	**0.67**	**−1.44**	0.49	**0.72**	**1.04**
1 versus 11	**−0.67**	0.17	0.01	**−1.67**	**0.91**	0.28	0.41
2 versus 3	0.19	0.32	−0.48	0.32	0.33	−0.06	**−0.73**
2 versus 4	**0.61**	0.25	**−1.18**	**0.78**	0.00	−0.47	−0.46
2 versus 5	0.20	**−0.52**	0.15	**0.54**	−0.33	0.35	−0.26
2 versus 6	**0.72**	0.15	**−0.97**	0.26	0.13	**−0.68**	−0.18
2 versus 7	0.05	0.09	−0.03	−0.02	0.31	0.02	−0.43
2 versus 8	**−1.72**	**−0.58**	0.42	**0.78**	**−1.42**	**1.20**	**0.89**
2 versus 9	−0.01	**1.45**	**−1.82**	0.27	0.47	**−0.51**	0.09
2 versus 10	−0.26	−0.08	0.02	0.34	0.09	0.03	−0.07
2 versus 11	0.35	0.28	**−0.64**	0.11	**0.51**	−0.41	**−0.70**
3 versus 4	0.43	−0.06	**−0.69**	0.46	−0.33	−0.40	0.26
3 versus 5	0.01	**−0.84**	**0.63**	0.22	**−0.67**	0.40	0.47
3 versus 6	**0.53**	−0.17	−0.49	−0.06	−0.20	**−0.62**	**0.55**
3 versus 7	−0.13	−0.22	0.45	−0.34	−0.03	0.07	0.30
3 versus. 8	**−1.90**	**−0.90**	**0.90**	0.46	**−1.76**	**1.26**	**1.62**
3 versus 9	−0.20	**1.13**	**−1.34**	−0.04	0.13	−0.45	**0.82**
3 versus 10	−0.44	−0.39	0.50	0.02	−0.25	0.09	**0.66**
3 versus 11	0.17	−0.04	−0.16	−0.21	0.18	−0.36	0.03
4 versus 5	−0.42	**−0.78**	**1.33**	−0.24	−0.33	**0.80**	0.20
4 versus 6	0.10	−0.11	0.21	**−0.52**	0.13	−0.22	0.29
4 versus 7	**−0.56**	−0.16	**1.15**	**−0.80**	0.31	0.47	0.03
4 versus 8	**−2.33**	**−0.84**	**1.60**	0.00	**−1.42**	**1.66**	**1.36**
4 versus 9	**−0.63**	**1.20**	**−0.65**	−0.50	0.47	−0.05	**0.56**
4 versus 10	**−0.87**	−0.33	**1.19**	−0.44	0.09	0.48	0.40
4 versus 11	−0.26	0.03	**0.53**	**−0.67**	**0.51**	0.04	−0.24
5 versus 6	**0.52**	**0.67**	**−1.12**	−0.28	0.46	**−1.02**	0.09
5 versus 7	−0.15	**0.62**	−0.18	**−0.56**	**0.64**	−0.33	−0.17
5 versus 8	**−1.91**	−0.06	0.27	0.24	**−1.09**	**0.86**	**1.15**
5 versus 9	−0.21	**1.97**	**−1.98**	−0.26	**0.80**	**−0.85**	0.35
5 versus 10	−0.45	0.45	−0.14	−0.20	0.42	−0.32	0.20
5 versus 11	0.16	**0.80**	**−0.80**	−0.43	**0.84**	**−0.76**	−0.44
6 versus 7	**−0.66**	−0.05	**0.94**	−0.28	0.17	**0.69**	−0.26
6 versus 8	**−2.43**	**−0.73**	**1.39**	**0.52**	**−1.56**	**1.88**	**1.07**
6 versus 9	**−0.73**	**1.30**	**−0.85**	0.01	0.33	0.17	0.27
6 versus 10	**−0.97**	−0.22	**0.99**	0.08	−0.05	**0.71**	0.11
6 versus 11	−0.36	0.13	0.33	−0.15	0.38	0.26	**−0.53**
7 versus 8	**−1.77**	**−0.68**	0.45	**0.80**	**−1.73**	**1.19**	**1.33**
7 versus 9	−0.07	**1.36**	**−1.79**	0.29	0.16	**−0.52**	**0.53**
7 versus 10	−0.31	−0.17	0.05	0.36	−0.22	0.01	0.37
7 versus 11	0.30	0.18	**−0.61**	0.13	0.20	−0.43	−0.27
8 versus 9	**1.70**	**2.03**	**−2.25**	−0.50	**1.89**	**−1.71**	**−0.80**
8 versus 10	**1.46**	**0.51**	−0.41	−0.44	**1.51**	**−1.17**	**−0.96**
8 versus 11	**2.07**	**0.86**	**−1.07**	**−0.67**	**1.94**	**−1.62**	**−1.59**
9 versus 10	−0.24	**−1.53**	**1.84**	0.06	−0.38	**0.53**	−0.16
9 versus 11	0.37	**−1.17**	**1.18**	−0.16	0.04	0.09	**−0.79**
10 versus 11	**0.61**	0.35	**−0.66**	−0.23	0.42	−0.44	**−0.64**
Gender							
M versus F	0.00	0.00	−0.24	0.11	0.16	−0.06	−0.02
Age							
C versus A	**−0.67**	−0.18	0.35	**−1.05**	0.28	**0.66**	**0.76**
C versus E	0.30	−0.49	0.11	−0.40	0.00	**0.73**	0.50
A versus E	0.37	−0.32	−0.24	**0.65**	−0.28	0.08	−0.26

Note

Country: 1 = Japan; 2 = Bangladesh; 3 = UK; 4 = Brazil; 5 = Taiwan; 6 = Italy; 7 = New Zealand; 8 = Iran; 9 = Cuba; 10 = Pakistan; 11 = French

Gender: M = male; F = female.

Age: C = children aged below 18 years; A = adults aged between 18 and 60 years; E = elderly aged over 60 years.

DIF contrasts >0.5 are **in bold**.

**TABLE 6:** Measurement invariance of confirmatory factor analysis (CFA)

	Fit statistics						
	*χ* ^2^	*df*	*p*‐value	CFI	TLI	RMSEA	SRMR
Country							
M1	1,146.162	154	<.001	0.985	0.978	0.067	0.052
M2	2,760.882	214	<.001	0.963	0.960	0.091	0.071
M3	1,748.925	194	<.001	0.977	0.973	0.075	0.059
M4	7,499.384	254	<.001	0.894	0.903	0.142	0.102
M5	2,372.629	204	<.001	0.968	0.964	0.086	0.066
Gender							
M6	1,288.944	28	<.001	0.983	0.974	0.076	0.053
M7	1,414.653	34	<.001	0.981	0.977	0.072	0.055
M8	1,511.912	40	<.001	0.980	0.979	0.069	0.056
Age							
M9	1,344.892	42	<.001	0.983	0.974	0.077	0.053
M10	1,806.105	54	<.001	0.977	0.973	0.079	0.055
M11	2,867.764	66	<.001	0.963	0.965	0.090	0.062

Note

M1 = configural model across ten countries; M2 = multigroup CFA across countries with factor loadings constrained equal; M3 = multigroup CFA across countries with factor loadings constrained equal, except for item loadings F2 and F3; M4 = multigroup CFA across countries with factor loadings and item intercepts constrained equal, except for item loadings F2 and F3; M5 = multigroup CFA across countries with factor loadings and item intercepts constrained equal, except for item loadings F2 and F3 and item intercepts F1, and F3 to F6; M6 = configural model across genders; M7 = multigroup CFA across genders with factor loadings constrained equal; M8 = multigroup CFA across genders with factor loadings and item intercepts constrained equal; M9 = configural model across age groups (<18 years; 18–65 years; and > 65 years); M10 = multigroup CFA across age groups (<18 years; 18–65 years; and > 65 years) with factor loadings constrained equal; M11 = multigroup CFA across age groups (<18 years; 18–65 years; and >65 years) with factor loadings and item intercepts constrained equal.

Abbreviations: CFI, comparative fit index; RMSEA, root mean square error of approximation; SRMR, standardized root mean square residual; TLI, Tucker‐Lewis index.

Table 7 presents the factor loadings and item intercepts of the FCV‐19S items across countries, gender and age groups in the constrained multigroup CFA models. Moreover, the latent FCV‐19S means showed that the Iranian participants had the highest levels of fear (effect size = 2.132) and the New Zealand participants had the lowest levels of fear (effect size = −1.435). The latent means are comparable to the observed FCV‐19S scores shown in Table 2. Additionally, male participants as compared with female participants had lower levels of fear (effect size = −0.251); children aged below 18 years (effect size = −0.527) and older people aged above 60 years (effect size = −0.193) as compared with young to middle‐aged adults aged between 18 and 60 years had lower levels of fear. The SEM concurred with the findings found in the multigroup CFA (Figure 1). Female participants as compared with male participants had higher levels of fear (standardized coefficient = 0.120); children and older people as compared with young to middle‐aged adults had lower levels of fear (standardized coefficients = −0.080 and −0.043, respectively).

**TABLE 7:** Factor loadings and item intercepts in the Fear of COVID‐19 Scale (FCV‐19S)

	Country											Gender		Age group		
	Bangladesh	UK	Brazil	Taiwan	Italy	NZ	Iran	Cuba	Pakistan	Japan	French	Male	Female	Children	Adults	Elderly
Loading																
F1	0.695	0.693	0.701	0.608	0.689	0.588	0.471	0.588	0.710	0.576	0.675	0.611	0.652	0.472	0.648	0.609
F2^a^	0.631	0.668	0.706	0.860	0.621	0.698	0.373	0.417	0.751	0.552	0.668	0.646	0.678	0.462	0.673	0.610
F3^a^	0.739	0.701	0.613	0.815	0.648	0.721	0.635	0.433	0.681	0.632	0.697	0.730	0.696	0.633	0.712	0.794
F4	0.727	0.742	0.720	0.757	0.726	0.808	0.738	0.690	0.749	0.661	0.739	0.701	0.704	0.541	0.723	0.719
F5	0.749	0.695	0.711	0.733	0.651	0.587	0.556	0.705	0.704	0.570	0.619	0.679	0.717	0.493	0.706	0.690
F6	0.659	0.727	0.807	0.751	0.857	0.811	0.673	0.640	0.668	0.681	0.819	0.749	0.705	0.739	0.731	0.750
F7	0.689	0.830	0.764	0.855	0.690	0.876	0.845	0.667	0.729	0.698	0.856	0.761	0.730	0.700	0.755	0.805
Intercept																
F1^b^	3.477	3.382	3.254	2.598	3.726	3.020	2.050	1.884	3.231	4.017	3.291	3.040	3.131	2.850	3.062	2.611
F2	3.355	2.982	2.759	2.608	2.816	3.021	4.263	2.568	3.096	2.890	2.787	2.912	2.952	2.563	2.928	2.407
F3^b^	2.214	2.420	2.004	2.432	2.122	2.954	2.924	1.150	2.283	2.366	2.497	2.063	1.898	2.155	1.902	1.922
F4^b^	2.389	2.576	2.419	2.464	2.491	2.968	3.155	1.510	2.615	3.759	2.442	2.340	2.268	2.149	2.255	2.033
F5^b^	3.303	3.115	2.661	2.593	2.838	2.859	2.119	2.135	3.059	2.886	2.794	2.825	2.883	2.520	2.831	2.508
F6^b^	2.164	2.310	2.125	2.395	2.453	3.005	3.656	1.181	2.164	2.404	2.498	2.065	1.875	2.428	1.883	1.751
F7	2.268	2.517	2.120	2.361	2.200	3.083	3.746	1.603	2.315	2.559	2.603	2.133	1.978	2.347	1.984	1.918
FCV‐19S	0.000	−0.743	−0.572	−0.807	−0.804	−1.435	2.132	0.300	−0.595	−0.847	−1.061	−0.251	0.000	−0.527	0.000	−0.193

Note

The loadings and intercepts were calculated using constrained models (M5 in Table 5 for country; M8 in Table 5 for gender).

^a^ Relaxed for factor loadings.

^b^ Relaxed for item intercepts.

## 4 DISCUSSION

In order to respond to the need of assessing mental health difficulties and associated behaviors (e.g. problematic use of the internet, suicidal thoughts, sleep problems, psychological distress and panic buying) among different populations during the COVID‐19 pandemic (Holmes et al., 2020; Lin, 2020; Pramukti et al., 2020; Taylor et al., 2020), the present study used datasets from ten countries to evaluate the measurement invariance and other psychometric properties of the Fear of COVID‐19 Scale (FCV‐19S; Ahorsu, Lin, Imani, et al., 2020; Ahorsu, Lin, & Pakpour, 2020). With the use of CFA, multigroup CFA, and Rasch analysis, the psychometric properties of the FCV‐19S were reaffirmed to be satisfactory and consistent with prior findings (Alyami et al., 2020; Pakpour, Griffiths, & Lin, et al., 2020; Sakib et al., 2020; Satici et al., 2020; Soraci et al., 2020; Tsipropoulou et al., 2020). More specifically, the single‐factor structure was confirmed in different ethnic populations (Bangladeshi, British, Brazilian, Taiwanese, Italian, New Zealander, Iranian, Cuban, Pakistani, Japanese and French), different genders and different age groups (child, young to middle‐aged adult, and older people). Moreover, full measurement invariance without substantial DIF was supported for the FCV‐19S across gender and age groups, but not across ethnic populations. Partial invariance with substantial DIF was observed for the FCV‐19S across ethnic populations. The latent scores of the FCV‐19S showed that the Iranians had the highest levels of fear of COVID‐19, whereas the New Zealanders had the lowest; females had greater fear of COVID‐19 than males; and young to middle‐aged adults had more fear of COVID‐19 than children and older people. The SEM model further echoed the findings from the latent score comparison.

Full measurement invariance across different ethnic groups was not supported for the FCV‐19S. This may be explained by the different impacts of COVID‐19 across these countries. For example, Iran reported high numbers of confirmed cases and deaths during the initial COVID‐19 pandemic and many Iranians believed false COVID‐19 information (Ahorsu, Lin, Imani, et al., 2020; Fazeli et al., 2020; Hashemi et al., 2020; Lin et al., 2020; Lin, Imani, et al., 2020). Moreover, Bangladesh failed to control the transmission rate because of lockdown mismanagement, while several countries (e.g. Italy, Cuba, United Kingdom and Pakistan) had a high transmission rate of COVID‐19 even though the government implemented strict regulations in infection control (e.g. closures of public activities) (Mamun et al., 2021).

In contrast, New Zealand and Taiwan had good strategies to eliminate the has an impact of COVID‐19 (Chang, Strong, et al., 2020; Cheng et al., 2020; Winter et al., 2020). Indeed, the COVID‐19‐related deaths in New Zealand were 22 on August 3, 2020 (Center for Systems Science and Engineering [CSSE], Johns Hopkins University, 2020) and seven in Taiwan on August 17, 2020 (Center for Systems Science and Engineering [CSSE], Johns Hopkins University, 2020), although the population sizes were relatively small in the two countries (approximately 4.9 million population in New Zealand and 23.6 million population in Taiwan). Moreover, both Taiwan and New Zealand have had relatively few COVID‐19 infection cases due to the good control in transmission. This may be because both countries are islands, which may have better border control than some other countries. Therefore, individuals living in different countries may have different feelings and perceptions because of the various COVID‐19 situations and related policies. Subsequently, measurement invariance cannot be supported based on the present study's findings.

Another explanation is cultural differences. Western people embrace individualism (Dubois & Beauvois, 2005; Harkness et al., 2000) and respect their freedom substantially. Therefore, whenever a behaviour is not prohibited by the legislation (e.g. wearing mask), Western people are not likely to violate their will to perform this behaviour. In contrast, Eastern people in collectivism cultures are prone to satisfy the community harmony. Therefore, even a simple behaviour such as wearing mask is not required by the laws, Eastern people are likely to perform this behaviour to align themselves to the society norm (King & Bond, 1985; Lin & Tsai, 2016; Tsai et al., 2015). Apparently, Westerners and Easterners have different attitudes toward hygiene behaviours (e.g. Westerners tend not to wear face masks) (Rieger, 2020) and the different attitudes may reflect the different interpretations of the FCV‐19S. Nevertheless, partial invariance of the FCV‐19S was supported across different ethnic populations. More specifically, Items F2 and F3 were not invariant across countries in their factor loadings; Items F1 and F3 to F6 were not invariant across countries in their item intercepts; and only Item F7 was entirely invariant across countries. Therefore, when researchers or healthcare providers wish to use the FCV‐19S to conduct comparisons across countries, they should be aware of the different weightings could occur for Items F2 and F3. Different initial rating scores could also occur for items F1 and F3 to F6.

Findings from both multigroup CFA and Rasch analysis generally supported the notion that the participants interpreted the FCV‐19S in a similar way irrespective of their gender and age. Further comparisons on the FCV‐19S between gender and age indicate that females had greater fear of COVID‐19 than males, and young to middle‐aged adults had greater fear than children and older people. The higher fear found among females can be explained by their higher sensitivity toward stress than males (Tolin & Foa, 2006; Vlassoff, 2007). With the high sensitivity toward stress (e.g. COVID‐19 pandemic), females are likely to develop greater fear than males.

Children as compared with young to middle‐aged adults showed lower levels of fear toward COVID‐19. This may be explained by the different perceptions of COVID‐19. As children are not like young to middle‐aged adults in encountering difficult challenges resulting from COVID‐19 (e.g. financial burden), children may not consider COVID‐19 a serious problem and therefore do not have high levels of fear toward COVID‐19 (Chen, Jyan, et al., 2020). Surprisingly, older people as compared with young to middle‐aged adults also had lower levels of fear toward COVID‐19. This finding may be seen as surprising given the fact that older people with COVID‐19 have higher mortality rates than young to middle‐aged adults (Dariya & Nagaraju, 2020; Moccia et al., 2020). However, it may be that older people may consider that they have little to lose as they have already had relatively long lives. Therefore, they may have made more preparations in relation to their own death than young to middle‐aged adults and subsequently lowered their fear of COVID‐19. However, additional evidence is needed to examine such speculation. Moreover, although the present findings showed a lower fear of COVID‐19 among children and older people, but healthcare providers and policymakers should not ignore the importance of mental well‐being in these two populations. More specifically, prior evidence has shown the need to tackle their mental health issues during COVID‐19, including increased psychological distress, sleep problems, and problematic use of the internet (Ahorsu, Lin, & Pakpour, 2020; Chen et al., in press; Chen et al., 2020).

In addition, different factor loadings were observed across countries, genders, and age groups (Table 7). More specifically, most of the factor loadings were strong and consistent (i.e. between 0.5 and 0.9) across the countries for all the seven FCV‐19S items, except for Items F1 (loading = 0.471) and F2 (loading = 0.373) in Iran. Therefore, Iranians might be interpreting words such as “afraid” and “uncomfortable” differently to individuals in other countries when evaluating their fear of COVID‐19. A similar pattern was observed among children as compared with the other two age groups. Items F1 (loading = 0.472) and F2 (loading = 0.462) were the lowest among the seven FCV‐19S items. Therefore, children when compared to the other two age groups appear to interpret the words “afraid” and “uncomfortable” less when considering fear of COVID‐19. In relation to gender, it appeared that both genders considered all the seven FCV‐19S items as of equal and strong importance when evaluating their fear of COVID‐19.

The major strength of the present study is the use of cross‐country data with a large sample size. The large sample size across different ethnic groups ensures the robustness of the dataset. Therefore, the present findings support the use of FCV‐19S in these ten countries. Another strength of the present study is the use of advanced psychometric testing. Multigroup CFA and Rasch analysis give different perspectives in the underlying testing theories (Lin et al., 2019). Therefore, the similar results found in both multigroup CFA and Rasch analysis ensure the good and stable properties of the FCV‐19S. Moreover, the comparisons of FCV‐19S latent scores made in the present study have considered the measurement issues and are much more robust than the comparisons of simple FCV‐19S observed scores (Lin et al., 2016; Vandenberg & Lance, 2000).

The present study's findings have some implications about the potential applications for nursing education, research, and practice in the clinical and community contexts. From the perspective of nursing education, it is worth introducing the timely and effective FCV‐19S to nursing students at all levels and areas. Nursing students may have initial ideas about the importance of assessing fear, and how fear can affect other aspects of health during COVID‐19 pandemic, and potential nursing actions that could be implemented into their day‐to‐day practice. Furthermore, nursing students will have the ability to generalize their learning concerning the FCV‐19S to other potential measures on fear of any infectious disease if there is another pandemic in the future. From the perspective of nursing research, the present study's findings assure nursing researchers that they can use FCV‐19S to assess fear of COVID‐19 efficiently and effectively among different populations. This information on fear can be used to support such researchers to investigate more deeply understudied psychological mechanisms during COVID‐19 pandemic. From the perspective of nursing practice, Registered nurses are encouraged to use the FCV‐19S to detect early the problems concerning fear of COVID‐19 among their patients. Consequently, appropriate nursing actions and treatments to reduce mental health issues or behaviour problems induced by fear of COVID‐19 could be immediately given.

The present study has the following limitations. First, all the data used in the present study were collected using convenience sampling methods. Therefore, the representativeness of the participants in each ethnic population is low. Also, the characteristics between the ten ethnic populations were not directly comparable and the comparisons of their FCV‐19S scores are somewhat biased. For example, the New Zealanders were aged below 20 years and the Taiwanese were aged above 50 years. With a 30‐year of difference in age, the comparison of FCV‐19S scores between New Zealanders and Taiwanese is obviously biased by age. Similarly, the Italian sample comprised extremely few males (8.0%) and the FCV‐19S score obtained was, therefore, more representative of females. Second, there were no other psychometric instruments used in the present study. Therefore, the present study could not examine how the FCV‐19S associated with other validated instruments and the concurrent validity of the FCV‐19S could not be concluded from the present findings. Third, the data collection periods were not comparable across the countries. With different policies and procedures to inhibit the spread of COVID‐19, and different numbers of cases and deaths, individuals' fear of COVID‐19 may have been different due to these factors. The changes of fear of COVID‐19 may thus be a potential confounding factor for the present study. Finally, given the importance of LGBT+ community, it is important to assess whether the FCV‐19S is invariant across “other” gender compared to male and female genders. Unfortunately, the present sample had too few participants of “other” gender to reliably calculate measurement invariance in this group. Future studies are, therefore, needed to recruit a large enough sample size of other gender to provide robust analysis on this important gender variant.

## 5 CONCLUSION

Based on the results of the present study, the FCV‐19S is a good psychometric instrument to assess fear of COVID‐19 during the pandemic period. Moreover, the use of FCV‐19S is supported in at least ten countries with satisfactory psychometric properties. However, only partial invariance rather than full measurement invariance of the FCV‐19S was supported across the ten ethnic populations. Therefore, future studies on FCV‐19S comparisons across different ethnic populations should be cautious with the measurement non‐invariance. Such studies may need to consider the use of multigroup CFA rather than simply summing up the FCV‐19S scores if they want to make the comparisons. Nevertheless, the full measurement invariance of the FCV‐19S was supported across gender and age groups. Therefore, future studies can reliably use the FCV‐19S to compare the fear of COVID‐19 between gender and age groups.

## CONFLICT OF INTEREST

The authors declare that they have no conflict of interest.

## AUTHOR CONTRIBUTIONS

All authors: Conceptualization, data analysis, final draft collection, data collection, data analysis, data interpretation, final discussion and review of the article.

## ETHICAL APPROVAL

All procedures performed in this study involving human participants were granted exemption by the Institutional Review Boards [University of Toulouse, France ("CER‐flash‐2020‐283"), Associazione Italiana Psicoterapia Cognitivo Comportamentale di Gruppo (Ringgold ID for this is 558,111), Hayatabad Medical Complex, Tohoku University Graduate School of Education's ethics committee (ID: 20‐1‐003), Institute of Allergy and Clinical Immunology of Bangladesh ethics board (i.e., IRBIACIB/CEC/03202005), the Research Ethics Committee (process number: 4.128.627), Ethics committee of Iryo Sosei University (reference number was #20‐02), the ethics committee of the Department of Psychology of the Universidad Central “Marta Abreu” de Las Villas, Taipei Medical University's ethical committee (TMU‐JIRB N202005044), Institute of Review Board (IRB) of the Jianan Psychiatric Center (JPC), Ministry of Health and Welfare Taiwan (IRB numbers: 20‐004), University of Otago Human Ethics Committee and the Ethics Committee of Qazvin University of Medical Sciences (IR.QUMS.REC.1398.375) and with the 1964 Helsinki declaration and its later amendments or comparable ethical standards.

## Data Availability

The authors elect not to share data. Research data are not shared for ethical and confidentiality reasons.
